# Facile synthesis of model polystyrene nanoparticles for nanoplastics research

**DOI:** 10.1016/j.mex.2026.104013

**Published:** 2026-06-25

**Authors:** Marta Férová, Petr Praus

**Affiliations:** a Department of Chemistry, University of Ostrava, 30. dubna 22, Ostrava, 701 03, Czech Republic; b Institut Environmental Technology, CEET, VSB - Technical University of Ostrava, 17. listopadu 15/2172, Ostrava-Poruba, 708 00, Czech Republic

**Keywords:** Nanoparticles, Nanoplastics, Polystyrene, Nanoprecipitation, Stability, Synthesis

## Abstract

This study presents a simple and inexpensive surfactant-free method for the synthesis of polystyrene (PS) nanoparticles that are suitable for use as model nanoplastics in environmental research. PS nanoparticles were prepared using a two-step process: polystyrene was dissolved in acetone and rapidly injected into heated deionised water (the Ouzo effect). An injection rate of 1 mL of the PS solution per 1.7 s led to the formation of the smallest nanoparticles with the average size of 40 nm. The temporal stability of the resulting nanosuspensions was also investigated. The average hydrodynamic sizes of the PS nanoparticles ranged from 40 to 44 nm over the period from 1 h to 265 d, and the corresponding zeta potentials ranged from -19.6 to -22.8 mV. The PS nanoparticles prepared using this procedure are free of stabilisers, easily reproducible, and scalable, making them a suitable reference material for further studies on the behaviour of nanoplastics.

• PS nanoprecipitation is driven by solvent polarity change.
• Ouzo-effect-induces nucleation and growth of nanoparticles tuneable by injection speed.
• Stabiliser-free, reproducible, and stable nanoplastic particles were obtained.

## Graphical abstract

**Figure:**
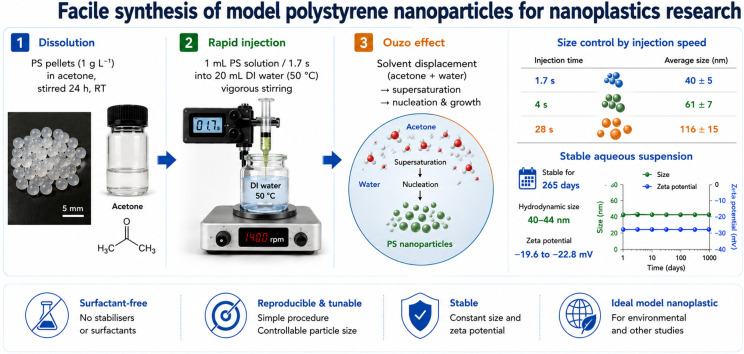

## Specifications table

**Table:** 

**Subject area**	Materials Science
**More specific subject area**	Nanoplastics
**Name of your method**	Synthesis of stable suspensions of polystyrene nanoparticles
**Name and reference of original method**	Nanoprecipitation
**Resource availability**	Polystyrene pellets (precursor), acetone, deionised water, hotplate with magnetic stirrer, Hamilton automatic syringe with 1 ml needle, and 25 mL glass vial (2 cm in diameter). Characterisation techniques: DLS, ELS, FTIR and UV spectroscopy, TEM, and cryo-TEM.

## Background

Research on environmental microplastics and nanoplastics has expanded rapidly in recent years. For fundamental studies, the use of well-defined artificial plastic microparticles and nanoparticles is essential for enabling precise monitoring of their environmental impacts. Owing to their extremely small size, nanoplastics can easily penetrate ecosystems and be ingested by a wide range of organisms, including aquatic species and humans. Notably, there is currently no universally accepted definition for the size of nanoplastics. Some authors define nanoplastics as plastic particles smaller than 1 µm, which is ten times larger than the commonly accepted 100 nm threshold for nanoparticle size (diameter), representing a thousand times greater particle volume.

Precipitation, or nanoprecipitation, first reported by Fessi et al. in 1989 [1] is a popular technique due to its simplicity, low energy consumption, easy implementation, tunability, reproducibility, and versatility. Precipitation in different solvents is known as the solvent displacement method or the Ouzo effect [[2], [3], [4]]. Plastic pellets made of polyethylene (PE), polypropylene (PP), polyvinyl chloride (PVC), and polystyrene have been produced by precipitation from xylene, cyclohexanone, and toluene solutions using ethanol or dimethyl sulfoxide as anti-solvents [[5], [6], [7]]. Lee et al. [8] reported the formation of PP nanoparticles by precipitating them from xylene solutions through ethanol addition. A similar method was employed by Cassano et al. [9], who generated PP nanoparticles (80–350 nm) by adding water to toluene solutions. However, the particles produced by these methods often exceed the 100 nm size typically used to define nanoplastics in the literature. True polymeric nanoparticles smaller than 100 nm were successfully synthesised [10] using a packed reactor filled with glass beads under UV irradiation.

Current research on nanoplastics has predominantly relied on commercially available plastic nanoparticles. However, these materials have several limitations. Commercial nanoparticles, which are often employed as standards for particle size measurements, are typically expensive and may contain trace amounts of surface-active agents (e.g., stabilisers or surfactants) added during synthesis. These additives can significantly modify the surface properties of nanoparticles and consequently influence the outcomes of experimental studies.

This study aims to synthesise plastic nanoparticles with a size of ≤ 100 nm that can serve as reference materials for the investigation and analysis of nanoplastics in environmental and other matrices. The resulting suspensions should be free of surfactants, have a defined chemical composition, and be easily prepared in the laboratory in any desired quantity at minimal cost. The proposed two-step procedure is based on the Ouzo effect and involves (i) the dissolution of PS pellets in acetone followed by (ii) the precipitation of PS nanoparticles in water. The obtained PS nanoparticles were characterised in terms of particle size, zeta potential, chemical composition, and morphology. In addition, the stability of the aqueous suspensions was studied because it is an important feature of reference materials. This study provides novel information on the practical feasibility of the precipitation of PS nanoplastics and their stability in aqueous suspensions.

## Method details

### Chemicals

Polystyrene pellets (No. 430,102, MW = 192 000) and acetone (Biosolve Chimie SARL, for LC-MS) were purchased from Sigma-Aldrich (Darmstadt, Germany). Deionised water was used in all experiments.

### Characterisation methods

#### Size and zeta potential measurement

The samples were characterised in terms of particle (hydrodynamic) size and zeta potential using a Malvern Zetasizer Advance Ultra (Malvern Instruments, UK) and the ZS Xplorer software version 4.0.0 (Malvern Instruments, UK).

#### FTIR spectroscopy

The Fourier-transform infrared (FTIR) (attenuated total reflection, or ATR) spectroscopy of PS samples was performed using a Thermo Nicolet 6700 (Thermo Fisher Scientific, USA). An FTIR spectrometer was equipped with a Smart iTR accessory at a spectral resolution of 8 cm^-1^ from 4000 to 400 cm^-1^.

#### UV spectroscopy

The UV spectra of PS samples were measured using a Cary 8454 UV–vis spectrometer (Agilent Technologies, USA) and processed using the ChemStation B.05.02 software (Agilent Technologies, USA). The samples were measured using a 10 mm quartz cuvette.

The DRS spectra were measured using a spectrometer Specord 250Plus (Analytik Jena, Germany). Before the analysis, virgin PS pellets were ground in a cryogenic mill (CryoMill, Retsch GmbH, Haan, Germany), and the granulometry was adjusted to <0.16 mm using an analytical sieve shaker AS200 (Restch, Haan, Germany) and a sieve (Preciselekt, Dolní Loučky, Czech Republic).

#### Transmission electron microscopy

The morphology of prepared PS nanoparticles was examined using transmission electron microscopy (TEM). The PS suspension was sonicated for 10 s, dropped onto a copper grid with a carbon film, and dried in air. TEM analysis was performed using a JEOL JEM 2100 microscope (JEOL Ltd., Japan) at an accelerating voltage of 200 kV. TEM images were analysed using the ImageJ 1.54 g software (National Institutes of Health, USA) to determine the sizes of the PS nanoparticles.

The morphology of prepared PS nanoparticles was also examined using cryogenic transmission electron microscopy (Cryo-TEM). A 4 µL drop of an undiluted sample was applied to a freshly plasma-cleaned TEM grid with a thin carbon layer and stained with 2% uranyl acetate for 30 s of sample incubation time and 1 min of staining time. The grid was loaded onto a Talos F200C (Thermo Scientific, USA) transmission electron microscope for imaging. The microscope was operated at 200 kV. Images were collected on a Ceta-16 M CMOS camera at a nominal magnification of 150,000 × with an underfocus of 1–3 µm.

### Polystyrene nanoparticles synthesis

**Fig. 1:**
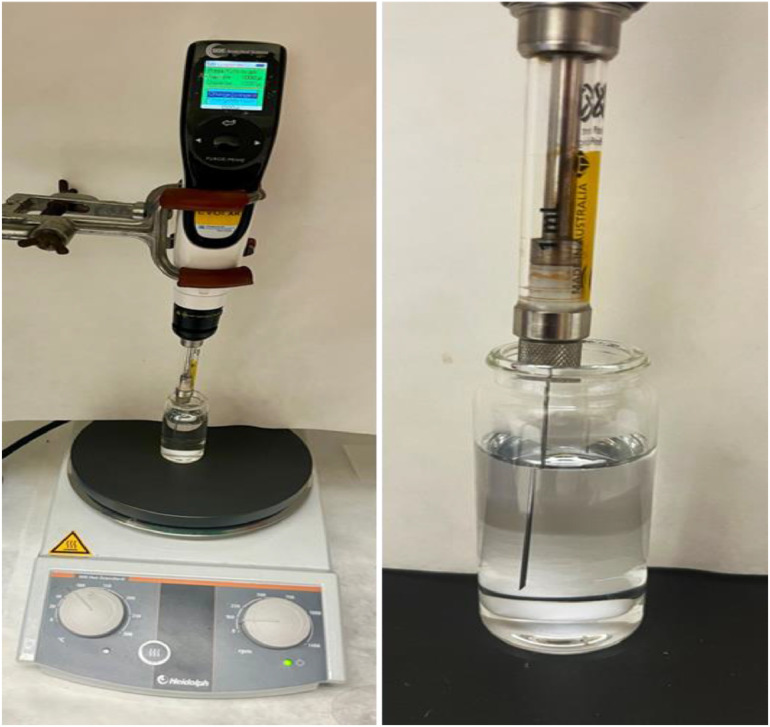
Experimental setup for the synthesis of PS NPs. Injection system (left) and detailed view of the dosing needle (right).

PS nanoparticles were prepared in a 25 mL glass vial (2 cm in diameter). Polystyrene pellets were dissolved in acetone to obtain a solution with a concentration of 1 g/L. After complete dissolution by stirring at room temperature, which occurred after at least 24 h, this solution was used as the precursor solution. First, 20 mL of deionised water was added to a vial and heated to 50 °C. Under vigorous stirring (a PTFE-coated magnetic stir bar, 10 mm length, 1400 rpm), 1 mL of the PS precursor acetone solution was rapidly injected into the vial using a Hamilton automatic syringe (eVol XR, with 1 mL syringe, SGE Analytical Science P/N 2910,035). The syringe needle was immersed near the bottom of the vial, perpendicular to the bottom and the solution was injected at the highest possible rate, as shown in Fig. 1. The injection was repeated (second volume directly after the first one, with only a brief pause to take in the next precursor) to add a total of 2 mL of the PS precursor to the water. After injecting the precursor, the resulting suspension was kept at 1400 rpm and 50 °C for approximately 10 s, then removed, capped, and left at room temperature to cool down. The sample was labelled PS-51. For comparison, two additional samples (PS-52 and PS-53) were prepared under identical conditions, except for the injection rate of the PS precursor into deionised water. In these cases, the injection rates were 1 mL per 4 s (PS-52) and 1 mL per 28 s (PS-53). Each sample was prepared in triplicate.

### Optimization of precipitation procedure

The optimisation of the PS nanoparticle synthesis method was based on a comprehensive study by Zhao et al. [4]. The precursor polystyrene concentration in acetone was set to 1 g/L. The temperature of the aqueous phase (deionised water), into which the precursor was injected, was maintained at 50 °C to avoid acetone evaporation during the experiments; the boiling point of acetone is 56 °C. Lowering the water temperature below 50 °C is not beneficial, because 50 °C is far below the glass transition temperature of polystyrene (Tg ≈ 100 °C). Under these conditions, particle formation is governed by acetone-water mixing kinetics rather than polymer segmental mobility. Reduced mass transfer at lower temperatures leads to fewer nucleation events and larger, more polydisperse nanoparticles.

**Fig. 2:**
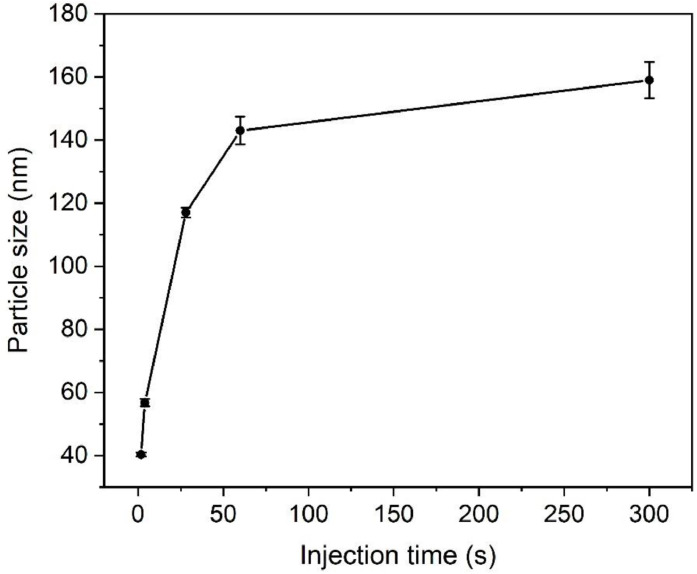
Size of PS-51 nanoparticles depending on the injection time of 1 mL of the PS precursor. The confidence intervals were calculated using *n* = 3.

Since the particle size decreases with increasing stirring rate [4], the precipitation process was investigated using a magnetic stirrer operated at its maximum rate of 1400 rpm. The PS precursor was injected as deep as possible below the surface of vigorously stirred deionised water using the 1 mL Hamilton automatic syringe. The injection time of 1 mL of the precursor was optimised for the values of 1.7 s, 4 s, 28 s, 60 s, and 300 s as demonstrated in Fig. 2. The injection was repeated to add a total of 2 mL of the PS precursor to the water.

The smallest NPs with an average size (z-avg) of 40.3 nm were obtained using the shortest possible injection rate: an injection of 1 mL per 1.7 s. This is in agreement with Weimarn´s law, because the highest supersaturation was achieved by the fastest injection. The further enlargement of the large particles can be explained by the agglomeration of smaller nanoparticles. An injection rate of 1.7 s was used for further experiments.

### Statistical calculations

Confidence intervals (CI) were calculated according to the equation

(1) $$CI=\bar{x}\;\pm \;SD\frac{t\left(\alpha ,n-1\right)}{\sqrt{n}}$$

where $\bar{x}$ is the arithmetic mean, *SD* is the standard deviation, *t* is the critical value from the Student’s t-distribution, and *n* is the number of measurements. The calculations were performed at a significance level α = 0.05.

## Method validation

### Characterization of nanoplastics by particle sizes and zeta potentials

**Fig. 3:**
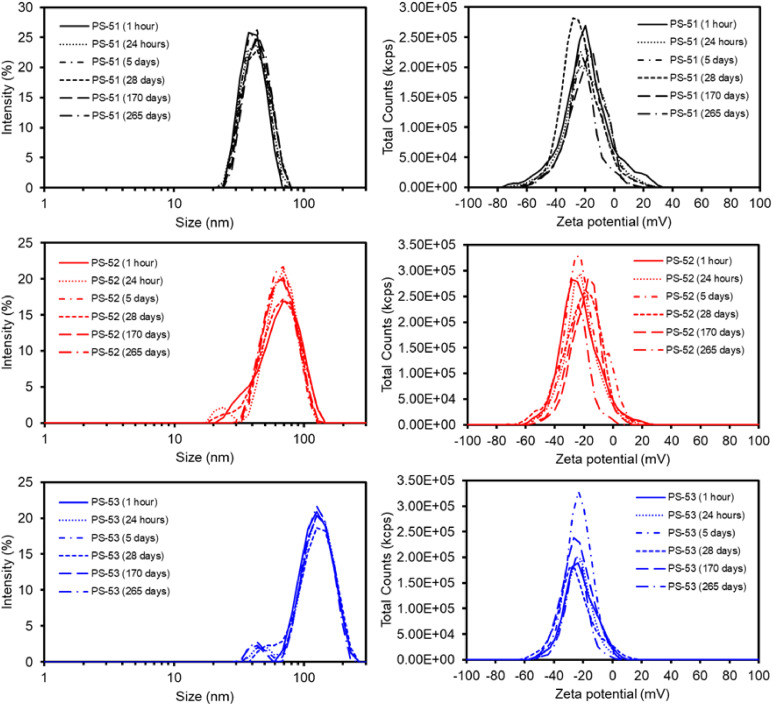
Measurement of the sizes and zeta potentials of PS nanoparticles from 1 h to 265 d.

**Fig. 4:**
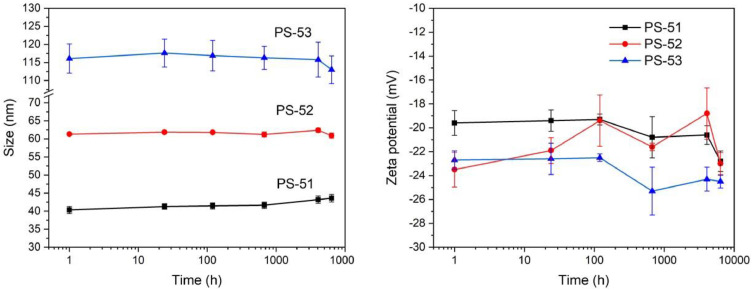
Sizes (left) and zeta potentials (right) of PS nanoparticles from 1 h to 265 d The confidence intervals were calculated using *n* = 30, 9, and 9 for PS-51, PS-52, and PS-53, respectively.

The measured sizes and zeta potentials of the polystyrene nanoparticles are presented in Fig. 3 and Table S1. The PS-51 samples analysed after 1 h, 1 d, 5 d, 28 d, 170 d, and 265 d exhibited narrow and unimodal size distributions, indicating their high temporal stability. The average particle sizes were approximately 40–44 nm, and the zeta potentials ranged from −19.6 to −22.8 mV. In contrast, the PS-52 and PS-53 samples prepared using slower injection rates displayed higher particle sizes. For PS-52, the average particle sizes varied from 61 to 62 nm, whereas for PS-53, they were 113 to 118 nm. The zeta potentials were slightly more negative, ranging from −19.4 to −23.5 mV for PS-52 and from −22.5 to −25.3 mV for PS-53. The particle sizes and zeta potentials as a function of time are shown in Fig. 4. It is evident that the PS nanoparticles were stable against agglomeration for 265 days. The observed sizes correlated well with the aforementioned relationship between particle size and supersaturation: the slower injection rates led to the lower supersaturation of polystyrene in water, thereby decreasing the supersaturation, which resulted in the formation of larger particles. Although the zeta potential of approximately −20 mV indicates that the system is on the verge of instability, the long-term stability of particle sizes over time demonstrates that the colloidal solution is highly stable. For particles around 40 nm, this may indicate that the kinetic energy of Brownian motion is sufficiently high to prevent particles from gradually agglomerating and sedimenting.

The DLS analysis enables several approaches to interpret particle size data. The particle sizes presented in Fig. 4 are based on intensity-weighted distributions. According to the theoretical background of DLS measurements, this type of analysis tends to yield larger apparent hydrodynamic sizes because it is directly based on the scattering intensity measured by the instrument. In contrast, Table S1 also includes the particle size distributions calculated by volume and number distributions. These were derived from intensity-based data using material-specific parameters for “Polystyrene latex” (refractive index: 1.59; absorption: 0.01), provided by the instrument software. The resulting modal particle sizes were 37–41 nm (by volume) and 33–37 nm (by number) for the sample PS-51; 49–57 nm (by volume) and 37–59 nm (by number) for the sample PS-52; and 99–125 nm (by volume) and 75–98 nm (by number) for the sample PS-53. All samples exhibited relatively narrow particle size distributions. The Di(90) parameter, defined as the hydrodynamic size below which 90% of the particles fall (based on intensity distribution), was 54–59 nm for PS-51, 90–101 nm for PS-52, and 176–187 nm for PS-53.

### Stability of nanoplastics suspensions

**Fig. 5:**
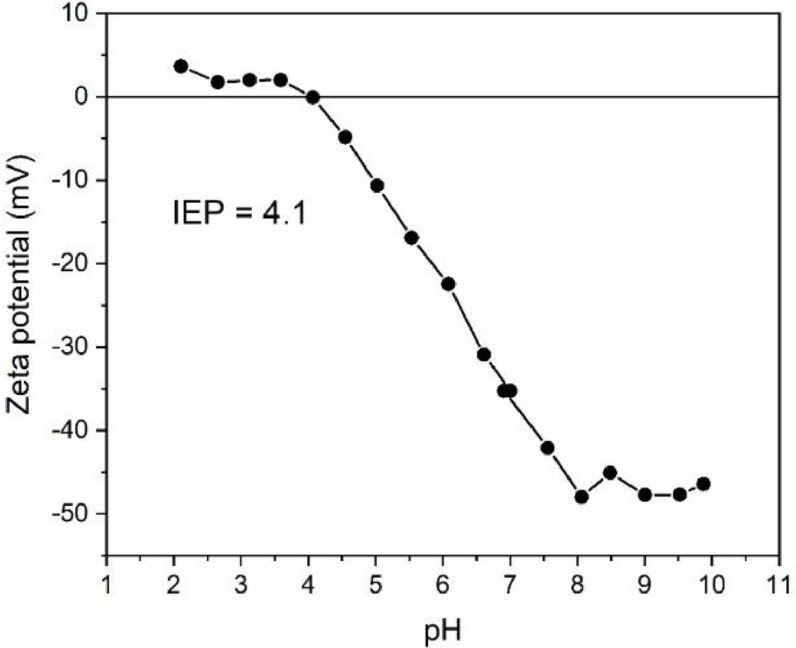
Increase in zeta potentials of PS-51 as a function of pH in the range of 2–10. IEP stands for isoelectric point.

Fig. 4 demonstrates the long-term stability of the PS nanoplastic suspensions, even in the absence of stabilising additives. This can be explained by the adsorption of OH^-^ ions on the hydrophobic PS surface. Theoretical [11] and experimental [12] studies have reported the preferential adsorption of OH^-^ ions compared to hydronium (H_3_O^+^) ions, as well as chloride and potassium ions. This was documented by measuring the zeta potentials against the pH of the suspensions; see Fig. 5. The isoelectric point was estimated to be 4.1, which is close to the value of 4.0 reported by Zimmermann et al. [12]. The negative surface charge of air bubbles, oil droplets, and hydrophobic solid surfaces was also experimentally confirmed by Beatttie et al. [13,14], Preočanin et al. [15], and others. However, the fundamental properties of water at hydrophobic interfaces, such as its orientation, the concentration of hydron and hydroxide ions, irregular hydrogen bonding, and the presence of strong electric fields, remain the subject of intense debate [16].

Fig. 5 also shows that the lowest zeta potentials of −50 mV can be reached at pH 8–10. Under these conditions, the surfaces of the PS nanoparticles were likely saturated with adsorbed OH^−^ ions. The pH value of PS-51 nanosuspensions was about 6 which corresponds to the zeta potential of −21 mV.

It should be noted that at pH = 6, deionised water also contains HCO_3_^-^ ions. For the equilibrium of CO_2_ in water, it can be found in textbooks that log c(HCO_3_^-^) = −11.3 + pH, which holds for a temperature of 25 °C, an ionic strength of 0, and a partial pressure of CO_2_ in the atmosphere of 30 Pa [17]. From this relationship, it follows that c(HCO_3_^-^) » 500 c(OH^-^). Therefore, the effects of HCO_3_^-^ and CO_3_^2-^ (at higher pH) naturally existing in water on the zeta potentials should be considered. The role of HCO_3_^-^ in charging water-hydrophobuc surfaces has been already reported by Yan et al. [18] and should be further studied experimentally and theoretically. However, this is beyond the scope of the current methodological study.

### Characterisation of nanoplastics by TEM

**Fig. 6:**
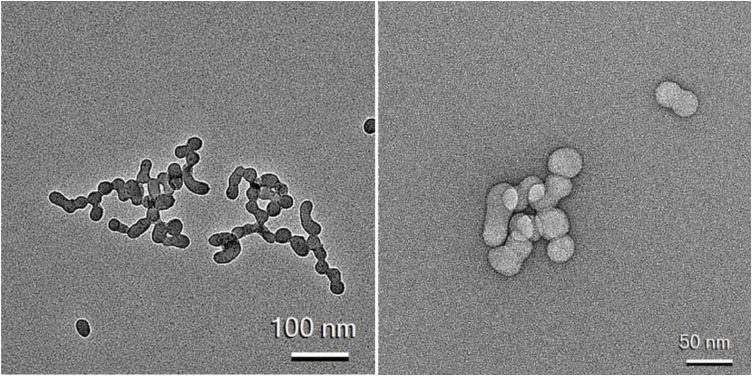
Cryo-TEM images of PS-51 nanoparticles with different resolutions.

The morphologies of the PS nanoparticles were examined using transmission electron microscopy. A representative cryo-TEM image of PS-51 is shown in Fig. 6. Several individual nanoparticles are visible, although many appeared to agglomerate into chain-like structures. Despite this agglomeration, individual particles could still be distinguished. It should be noted that the nanoparticles were subjected to specific sample preparation procedures for the TEM analysis, and the resulting images provide only an approximate representation of their morphology in aqueous suspensions because the samples are frozen and have a tendency to agglomerate than in aqueous systems. For comparison, the common TEM images are shown in Fig. S2. The greater tendency to agglomeration caused by drying before the TEM imaging is obvious. However, the individual particles are still distinguishable.

The sizes of the PS nanoparticles were also determined by analysing the common TEM images using the ImageJ software (Fig. S2). Individual nanoparticles were identified, and their areas were approximated as circles to calculate their corresponding sizes. A histogram of the particle size distribution is presented in Fig. S3, and the basic statistical parameters are summarised in Table 1. Based on skewness and kurtosis analyses, the particle size distribution did not follow a normal distribution. Therefore, both the median and average values of 35 and 36 nm, respectively, were calculated. These values are similar and in good agreement with the DLS average size of 40–43 nm (Table. S1). The particle sizes determined by TEM represent the nanoparticles in the dried state, which may differ from the hydrodynamic particle sizes determined by DLS.

**Table 1:** Basic statistics of PS-51 nanoparticles.

Parameter	Value	Parameter	Value
Number of nanoparticles	502	Maximal size (nm)	66
Average size (nm)	36	Range (nm)	50
Median size (nm)	35	Skewness	0.437
Minimal size (nm)	16	Kurtosis	3.36

### Characterisation of nanoplastics by FTIR spectroscopy

**Fig. 7:**
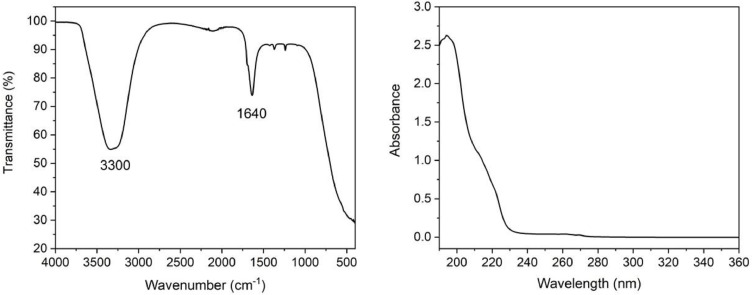
FTIR (left)and UV (right) spectra of PS nanoplastics.

The FTIR spectrum in Fig. 7 exhibits two prominent absorption bands: a broad band around 3300 cm⁻¹ and a sharper band at approximately 1635 cm⁻¹. The broad band is attributed to O-H stretching vibrations of adsorbed water [16], likely due to the large surface area of the PS nanoparticles. The band at 1630 cm⁻¹ corresponds to the C=C stretching vibrations in the aromatic rings of polystyrene [19,20]. The increase in absorption from 1000 to 400 cm^-1^ can be attributed to the out-of-plane bending vibrations of the styrene ring [20]. For comparison, the FTIR spectrum of virgin PS pellets was also recorded, as shown in Fig. S4. While the spectrum of PS pellets corresponds to the bulk structure, the spectrum of PS nanoplastics corresponds to their dominating surface with adsorbed water.

### Characterisation of nanoplastics by UV spectroscopy

The UV spectrum (Fig. 7) was measured after removing acetone residues from the NPs suspension by dialysis through a cellulose ester dialysis membrane with a volume of 10 mL and a molecular weight cut-off of 100–500 Da. The sample was dialysed four times in 1 L of deionised water. For comparison, Fig. S5 shows the UV spectrum of PS NPs suspension with acetone. The absence of acetone was also confirmed by gas chromatography, in which no acetone was detected (a detection limit of 0.8 mg/L). It should also be noted here that our DLS measurements confirmed that the removal of acetone from water did not cause particle aggregation, as shown in Fig. S1.

The UV spectra of PS nanoplastics exhibited an overall profile similar to that reported by Ducoli et al. [21], who observed comparable spectral features for fragmented polystyrene nanoparticles (average size of 100 nm). In their study, the spectra of particles of different sizes were compared, and a shift toward lower wavelengths was associated with a decrease in particle size. A direct comparison of band positions is not possible in this case because the band of our nanoparticles probably lies outside the measured range (< 200 nm). However, the right-hand shoulder of the band is comparable: in the literature, this shoulder begins to rise more steeply at approximately 300 nm. In our measurements, a pronounced increase was observed at the wavelengths of 230 nm and below, indicating that the nanoparticles have sizes smaller than 100 nm, which is consistent with the DLS and TEM data. The authors in [22] reported a spectrum with absorbance up to 270 nm. Wang et al. [23] worked with PS NPs of 80 nm in size, and their spectra show slightly increased absorbance below 275 nm and a higher increase in absorbance below approximately 237 nm, which is entirely comparable to our results and also leads to the conclusion that the particles prepared in this case are of smaller size.

To exclude light scattering on the suspensions of PS NPs, the UV spectra were measured at three positions of a cuvette with the sample: at a light source (position 1), at a detector (position 2), and between them (position 3). All three measurements (Fig. S6) overlapped, indicating that scattering on PS NPs is minimal and does not affect the measured spectra. Additionally, for comparison, a UV spectrum of a powder of milled PS virgin pellets was measured by diffuse reflectance spectroscopy (DRS) (Fig. S7).

## Limitations

The PS nanoparticles with the average size of 40 nm were obtained at the injection rate of 1 mL per 1.7 s. Shorter injection times, which would likely result in higher supersaturation and thus leading to the formation of smaller nanoparticles, were not technically feasible. Transmission electron microscopy was performed on dried samples after evaporation or cooling to cryogenic temperatures of the PS suspensions, therefore, the obtained images provide only an approximate representation of particle morphology in aqueous suspensions. Residual acetone (≈ 10 vol%, corresponding to a 2 mL injection into 20 mL of water) was not quantified over time. However, its concentration is expected to remain constant, so any potential influence would be systematic rather than time-dependent. It was demonstrated by DLS that the acetone content does not affect the size of PS particles in water.

## Ethics statements

None.

## CRediT authorship contribution statement

**Marta Férová:** Methodology, Investigation, Visualization, Writing – original draft, Writing – review & editing. **Petr Praus:** Conceptualization, Formal analysis, Visualization, Writing – original draft, Writing – review & editing.

## Declaration of competing interest

The authors declare that they have no known competing financial interests or personal relationships that could have appeared to influence the work reported in this paper.

## Data Availability

Data will be made available on request.

## Appendix Supplementary materials

Image, application 1
