# Supplementary material for: Facile synthesis of model polystyrene nanoparticles for nanoplastics research
Source: MethodsX. 2026 Jun 25;17:104013. doi: 10.1016/j.mex.2026.104013 (PMC13325323; doi:10.1016/j.mex.2026.104013)
Supplement: Supplementary file 1 [file mmc1.docx]

| **Subject area** | Materials Science |
| --- | --- |
| **More specific subject area** | Nanoplastics |
| **Name of your method** | Synthesis of stable suspensions of polystyrene nanoparticles |
| **Name and reference of original method** | Nanoprecipitation |
| **Resource availability** | Polystyrene pellets (precursor), acetone, deionised water, hotplate with magnetic stirrer, Hamilton automatic syringe with 1 ml needle, and 25 mL glass vial (2 cm in diameter). Characterisation techniques: DLS, ELS, FTIR and UV spectroscopy, TEM, and cryo-TEM. |

**Supplementary material**

Table S1. Sizes and zeta potentials of PS nanoparticles

|  | Time | Z-avg (nm) | SD (nm) | In Range (%) | PDI | Di(90) (nm) | Mode by intensity (nm) | Mode by vol. (nm) | Mode by number (nm) | Zeta potent. (mV) | SD (nm) |
| --- | --- | --- | --- | --- | --- | --- | --- | --- | --- | --- | --- |
| PS-51 | 1 h | 40.3 | 2.7 | 97.10 | 0.08336 | 54.2 | 41.3 | 36.9 | 33.4 | -19.6 | 2.8 |
|  | 1 d | 41.3 | 2.0 | 97.09 | 0.08139 | 57.1 | 42.9 | 37.8 | 33.8 | -19.4 | 2.4 |
|  | 5 d | 41.5 | 2.3 | 96.99 | 0.08576 | 55.9 | 42.3 | 37.9 | 34.2 | -19.3 | 1.2 |
|  | 28 d | 41.6 | 2.4 | 97.34 | 0.07337 | 59.0 | 43.8 | 37.8 | 33.2 | -20.8 | 4.6 |
|  | 170 d | 43.2 | 2.7 | 97.12 | 0.08304 | 58.6 | 44.4 | 39.6 | 35.7 | -20.6 | 2.1 |
|  | 265 d | 43.6 | 2.8 | 97.19 | 0.08373 | 58.8 | 45.0 | 40.7 | 37.0 | -22.8 | 2.3 |
| PS-52 | 1 h | 61.3 | 0.4 | 97.34 | 0.1512 | 101 | 68.7 | 52.4 | 41.8 | -23.5 | 1.9 |
|  | 1 d | 61.9 | 0.4 | 96.97 | 0.1361 | 96.8 | 69.2 | 56.9 | 55.8 | -21.9 | 1.4 |
|  | 5 d | 61.8 | 0.1 | 97.13 | 0.1463 | 91.5 | 66.9 | 57.0 | 48.9 | -19.4 | 2.8 |
|  | 28 d | 61.2 | 0.9 | 97.03 | 0.1496 | 101 | 68.9 | 49.2 | 36.5 | -21.6 | 0.4 |
|  | 170 d | 62.4 | 0.8 | 97.29 | 0.1295 | 96.9 | 67.6 | 53.2 | 58.9 | -18.8 | 2.8 |
|  | 265 d | 60.9 | 0.9 | 97.3 | 0.1274 | 90.4 | 65.4 | 55.6 | 48.1 | -23.0 | 1.2 |
| PS-53 | 1 h | 116 | 5 | 96.56 | 0.1323 | 187 | 130 | 113 | 88.5 | -22.7 | 1.0 |
|  | 1 d | 118 | 5 | 96.71 | 0.1320 | 183 | 134 | 125 | 88.5 | -22.6 | 1.7 |
|  | 5 d | 117 | 6 | 96.76 | 0.1287 | 183 | 132 | 122 | 98.2 | -22.5 | 0.4 |
|  | 28 d | 116 | 4 | 96.76 | 0.1302 | 184 | 127 | 99.3 | 74.8 | -25.3 | 2.6 |
|  | 170 d | 116 | 6 | 96.48 | 0.1275 | 183 | 133 | 119 | 90.9 | -24.3 | 1.3 |
|  | 265 d | 113 | 5 | 96.36 | 0.1347 | 176 | 130 | 122 | 86.5 | -24.5 | 0.7 |

Note: SD – standard deviation.


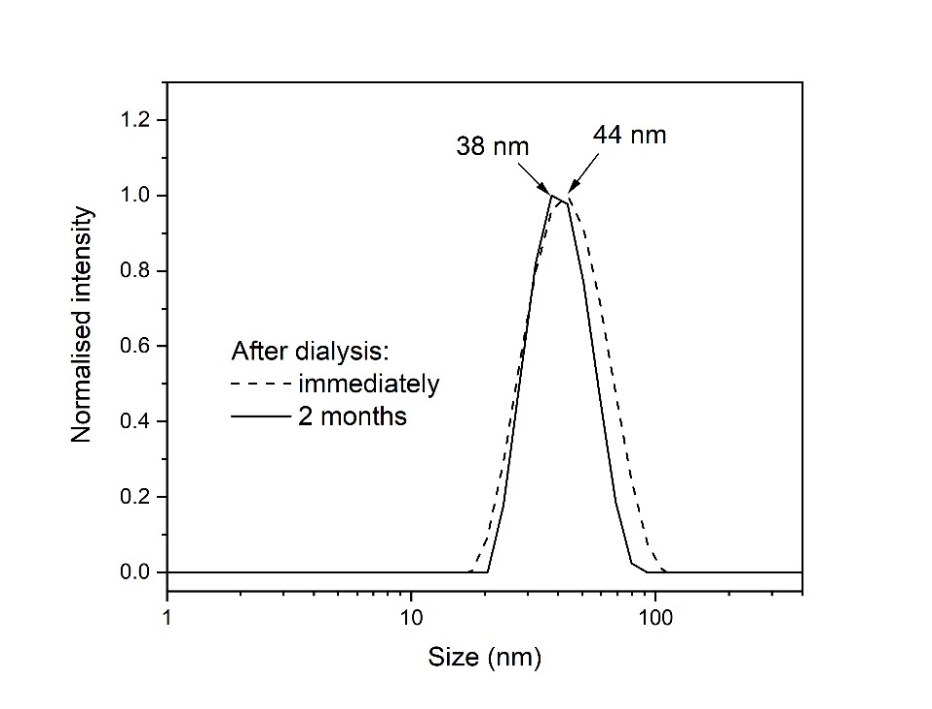


Fig. S1 Measurement of the sizes of PS-51 nanoparticles immediately and 2 months after dialysis.


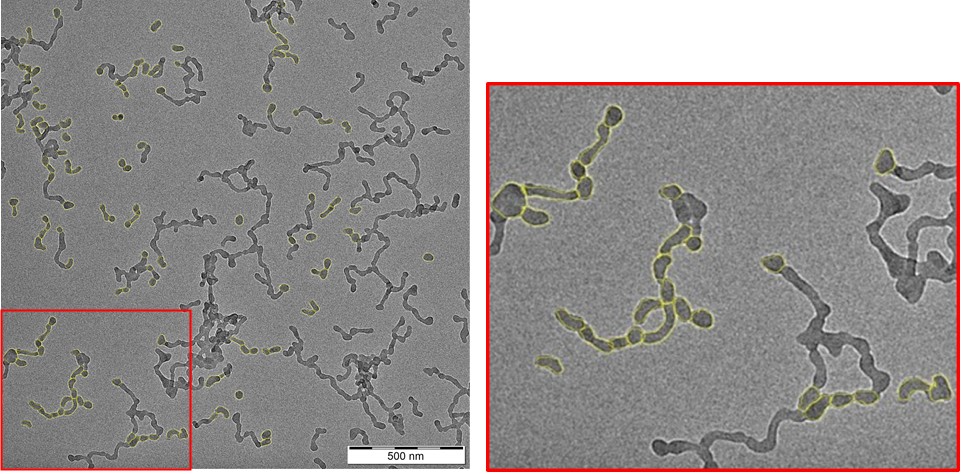


Fig. S2 Overall TEM image (left) of PS-51 nanoparticles, selected detail (right) of this TEM image.


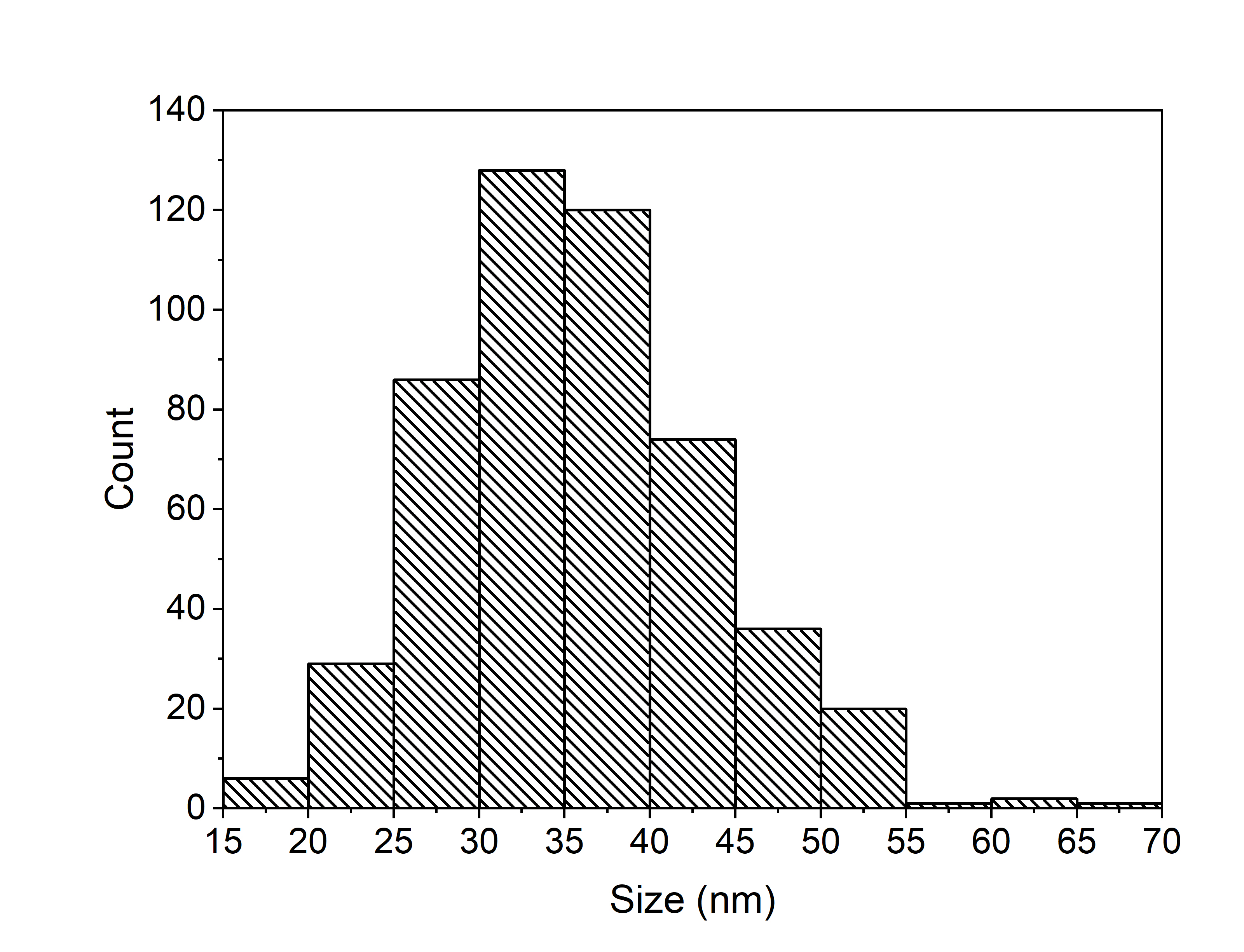


Fig. S3. Particle size distribution obtained from 502 particles identified in TEM images.


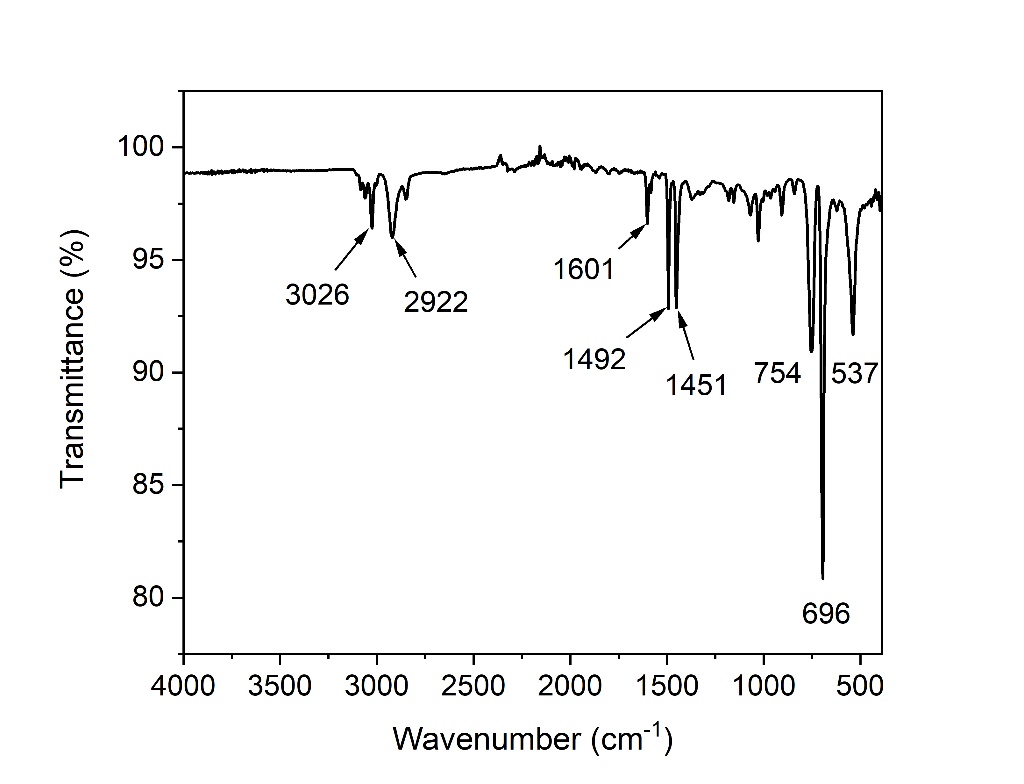


Fig. S4. FTIR spectrum of PS virgin pellets. The bands of 3026 and 2922 cm^-1^ present the C-H stretching vibrations. There are bands corresponding to the aromatic C=C stretching vibrations located at 1601, 1492, and 1451 cm^-1^. The two intense bands at 754 and 696 cm^-1^ can be explained by out-of-plane bending C-H vibrations and 537 cm^-1^ can be attributed to the deformation of the styrene ring.


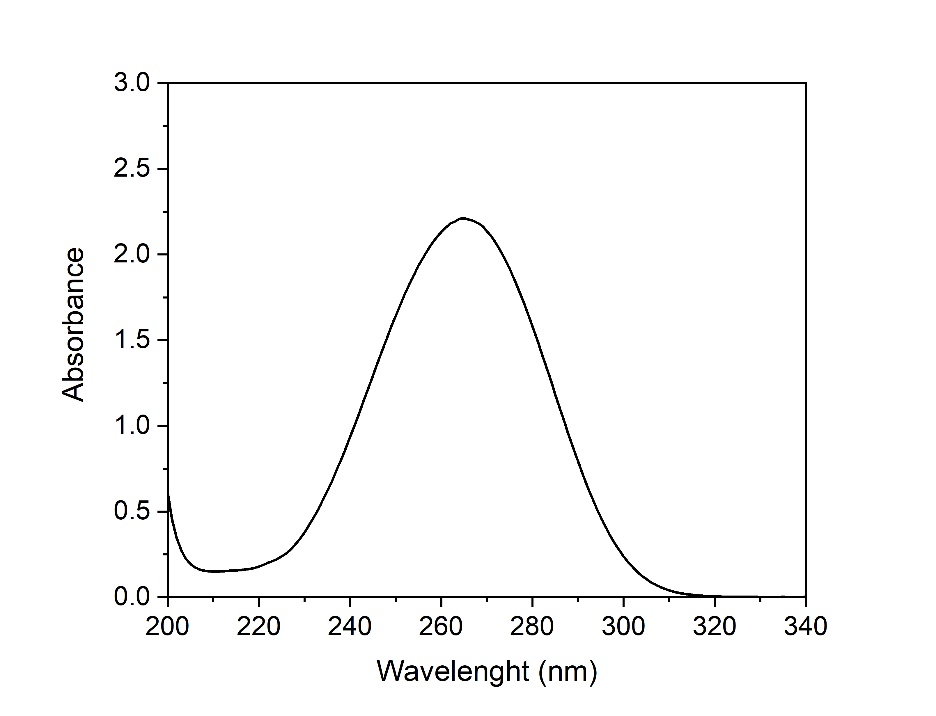


Fig. S5. UV spectrum of a PS-51 aqueous suspension with acetone.


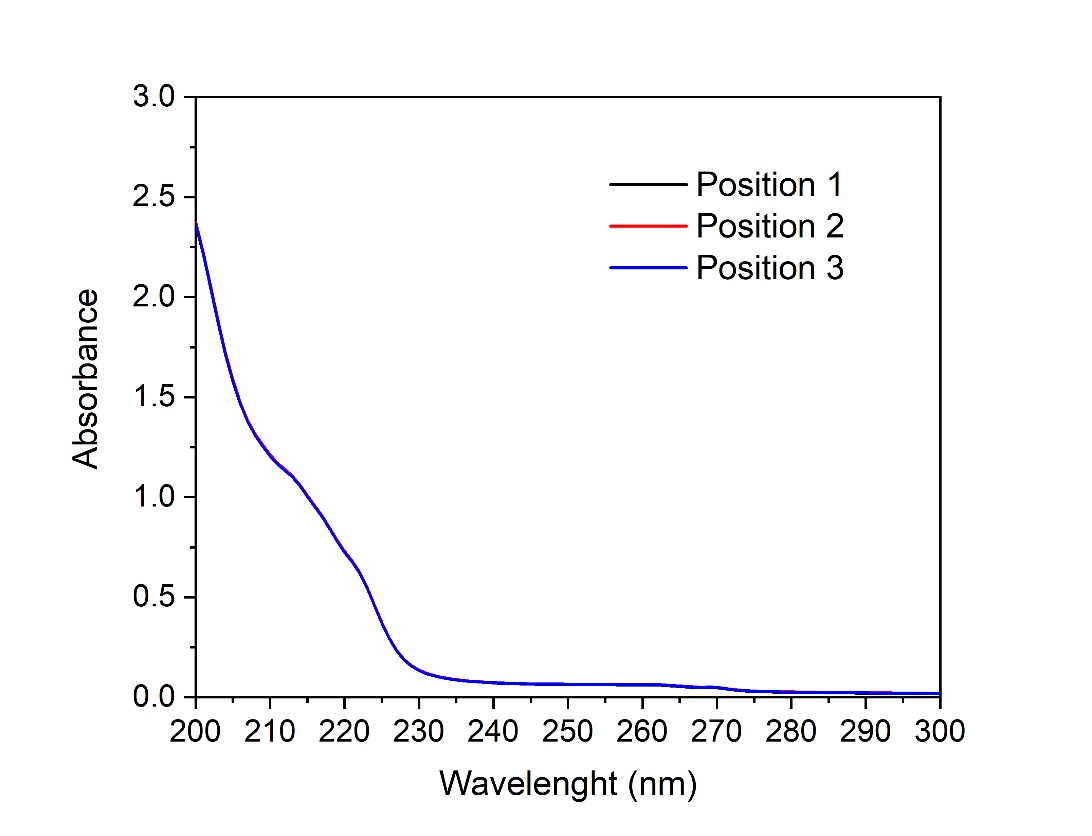


Fig. S6. UV spectra of PS-51 nanoparticle suspensions at three different cuvette positions.


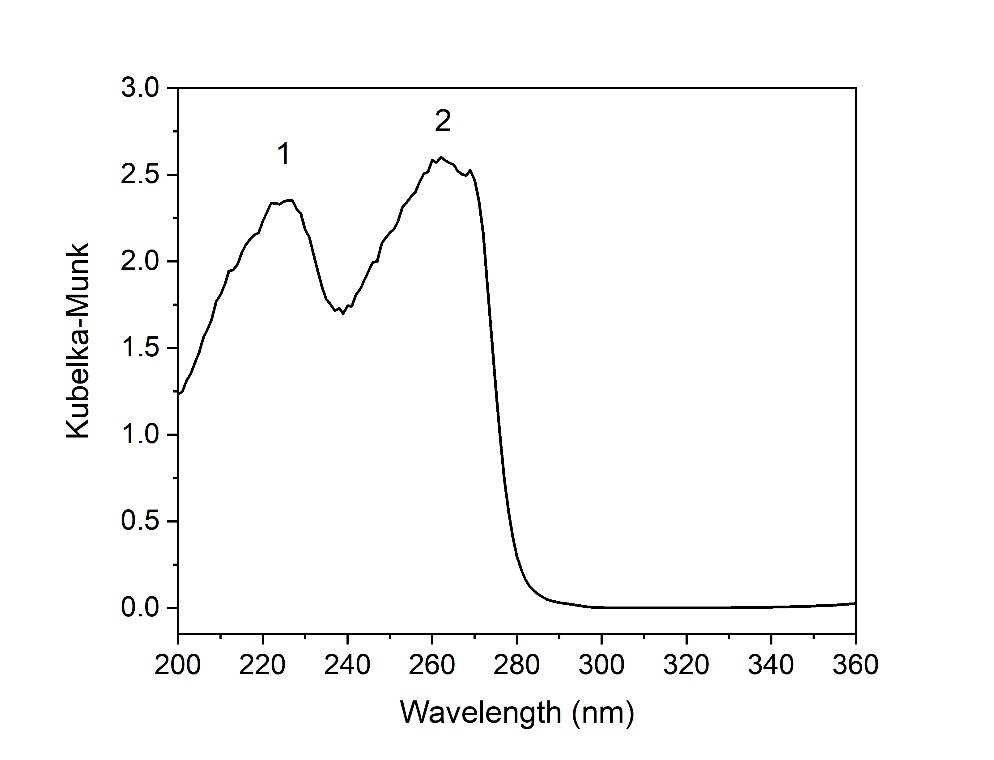


Fig. S7. UV spectra of PS virgin pellet powder (by DRS). The bands centred at approximately 225 nm (band 1) and 260 nm (band 2) indicate the characteristic electronic transitions of the benzene ring in polystyrene.
